# Effectiveness of the Dual GIP/GLP1-Agonist Tirzepatide in 2 Cases of Alström Syndrome, a Rare Obesity Syndrome

**DOI:** 10.1210/clinem/dgaf258

**Published:** 2025-04-30

**Authors:** Moritz Ferch, Isabel Peitsch, Alexandra Kautzky-Willer, Susanne Greber-Platzer, Albert Friedrich Stättermayer, Michael Krebs, Thomas Scherer

**Affiliations:** Division of Endocrinology and Metabolism, Department of Medicine III, Medical University of Vienna, Vienna 1090, Austria; Division of Endocrinology and Metabolism, Department of Medicine III, Medical University of Vienna, Vienna 1090, Austria; Division of Endocrinology and Metabolism, Department of Medicine III, Medical University of Vienna, Vienna 1090, Austria; Division of Pediatric Pulmonology, Allergology and Endocrinology, Department of Pediatrics and Adolescent Medicine, Medical University of Vienna, Vienna 1090, Austria; Division of Gastroenterology and Hepatology, Department of Medicine III, Medical University of Vienna, Vienna 1090, Austria; Division of Endocrinology and Metabolism, Department of Medicine III, Medical University of Vienna, Vienna 1090, Austria; Division of Endocrinology and Metabolism, Department of Medicine III, Medical University of Vienna, Vienna 1090, Austria

**Keywords:** Alström syndrome, tirzepatide, glucagon-like peptide 1, glucose-dependent insulinotropic polypeptide, GIP/GLP1 agonist, incretin

## Abstract

**Context:**

Tirzepatide, a dual glucose-dependent insulinotropic peptide/glucagon-like peptide 1 (GIP/GLP1) receptor agonist, was recently approved for type 2 diabetes and weight management. Alström syndrome (AS) is a rare, genetic, multisystemic disorder, characterized by cone-rod dystrophy, progressive hearing loss, obesity, and diabetes with profound insulin resistance due to marked hyperphagia.

**Objective:**

Here we highlight the potential of tirzepatide as a novel therapeutic option for improving glycemic outcomes, metabolic dysfunction–associated steatotic liver disease (MASLD), and effectively reducing body weight in individuals with AS.

**Methods:**

We present the first 2 reported cases of people living with AS treated with tirzepatide.

**Results:**

Two individuals with AS, previously treated with semaglutide, received tirzepatide at our clinic. The first, a 23-year-old man with 18 months of treatment, experienced a weight loss of −28 kg (113.6 to 83 kg, −26.9%); glycated hemoglobin A_1c_ decreased by −0.4% (6.7% to 6.3%), with considerable reductions in daily insulin doses of −96 IU/day (−83%; 58 to 20 IU insulin glargine and 58 to 0 IU prandial insulin), while maintaining oral antidiabetics. Hepatic steatosis, with a previous fat fraction of 20%, resolved as confirmed by magnetic resonance imaging (MRI). The second, a 20-year-old man with previously well-controlled diabetes, was followed up for 9 months and showed a weight reduction of −9.5 kg (132 kg to 122.5 kg; −7.2%) with a reduction of hepatic lipid content from 21% at the latest MRI to 11% after approximately 3 months of therapy.

**Conclusion:**

Tirzepatide shows great effectiveness with regard to body weight, MASLD, and insulin resistance in AS. Follow-up studies with larger cohorts have to be performed to confirm these findings.

Tirzepatide (Mounjaro^®^), a dual glucose-dependent insulinotropic peptide/glucagon-like peptide 1 (GIP/GLP1) receptor agonist, was recently approved for diabetes management as well as weight reduction for nondiabetic individuals with obesity or overweight and weight-related health problems.

Alström syndrome (AS) is a rare, genetic, multisystemic disorder caused by biallelic pathogenic variants in the *ALMS1* gene, which is characterized by a constellation of symptoms including cone-rod dystrophy and hearing loss, as well as obesity and type 2 diabetes due to hyperphagia. Management of diabetes in AS can be challenging due to extreme insulin resistance and poor glycemic control (1). GLP1 agonists have emerged as promising therapeutic options for the treatment of obesity and type 2 diabetes in general obesity (2); however, in cases of AS the effects were only moderate with a mean weight loss of 6% (−5.4 kg) (3). Furthermore, a clinical trial targeting hyperphagia with the melanocortin-4 receptor agonist setmelanotide failed to show significant effects in AS (4).

To date, no reports on the use of the novel dual GIP/GLP1 agonist tirzepatide on individuals with this rare obesity syndrome have been published. These cases highlight the potential of tirzepatide as a novel, more potent therapeutic option for improving glycemic outcomes, metabolic dysfunction–associated steatotic liver disease (MASLD), and reducing body weight as well as hyperphagia in individuals with AS.

## Materials and Methods

### Case 1

**Figure 1.**
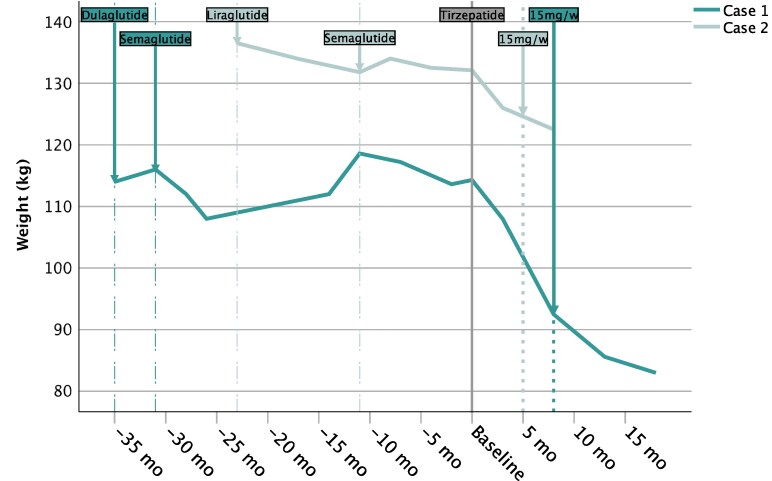
Weight trajectories during previous treatment with glucagon-like peptide 1 (GLP1) mono-agonists and during follow-up examinations since the start of tirzepatide (baseline) for both cases. Arrows on dashed lines indicate treatment start of the respective GLP1 mono-agonist for the respective case. All GLP1 agonists were uptitrated according to standard titration regimens to maximum dose. After initiation of tirzepatide (baseline, continuous line), both patients demonstrated accelerated weight loss during their respective 18- (case 1) and 8- (case 2) month follow-up periods on tirzepatide. Arrows on dotted lines indicate the time point when reaching the 15 mg/week maintenance dose of tirzepatide. Case 1 patient had to pause tirzepatide for approximately 6 weeks prior the latest follow-up due to temporary unavailability of the drug. No weight regain occurred during that time.

The individual was diagnosed with AS at age 12 after congenital retinal dysplasia advanced to complete amaurosis and auditory impairment. At that time, he suffered from type 2 diabetes mellitus (onset at age 9) and hepatic steatosis with early onset of severe obesity (99.6th percentile; *Z* score: 2.69). He was in ophthalmologic and otologic care since early childhood for photophobia, visual development retardation with congenital retinal dystrophia, and hearing impairment (onset at age 4) respectively and in pediatric specialist care since age 10. Conventional therapeutic approaches including physical exercise and dietary counseling, psychological and social-service support were futile, with persisting obesity (97.3th percentile, *Z* score: 1.92 at 11 years) and diabetes progressing to insulin-dependency at age 12, followed by multiple hospitalizations due to severe hyperglycemia prior to transitioning to adult care. At age 19, with a weight of 115 kg (body mass index [BMI] 42.8 kg/m^2^) and glycated hemoglobin A_1c_ (HbA_1c_) of 10.0%, semaglutide was started and uptitrated to 1 mg once weekly with an initial weight loss of −7 kg (BMI 40.2 kg/m^2^), and improvements in glucose control (HbA_1c_ 7.5%), rebounding to 119 kg (BMI 44.1 kg/m^2^) after 21 months (Fig. 1).

At the time of initiation of tirzepatide, the patient was under maximum oral antidiabetic medication (metformin 1000 mg twice a day, dapagliflozin 5 mg twice a day, pioglitazone 45 mg once a day, semaglutide 1 mg once weekly) as well as high doses of basal (58 IE) and prandial insulin (58 IE) with a totaling insulin dose of approximately 1 IU/kg body weight, achieving an HbA_1c_ of 6.7%. Growth hormone therapy at a dose of 0.2 mg daily was established at age 19 due to growth hormone deficiency (insulin-like growth factor-1: 88 ng/mL; reference range: 137-461 ng/mL) and reports on positive effects on hepatic steatosis (5). Growth hormone therapy was continued throughout. He was further on a stable dose of transdermal testosterone and oral levothyroxine due to hypogonadism and hypothyroidism, respectively. For further baseline characteristics, see Table 1.

**Table 1.** Baseline characteristics and follow-up data at latest time point of both patients

	Case 1 baseline	Follow-up (18 months on tirzepatide)	Case 2 baseline	Follow-up (9 months on tirzepatide)
Pathogenic variants (*ALMS1*)	c.6430C > T c.4150dupA		c.3132_3133delAC (homozygote)	
Diabetes mellitus type 2	yes		Yes	
MASLD	yes		Yes	
Hypogonadism	yes		Yes	
Hypothyroidism	yes		Yes	
Age, y	21	23	20	20
Sex	Male		Male	
BMI (kg/m^2^)	42.2	30.9	46.3	42.9
Weight, kg	113.6	83.0	132.1	122.5
Fasting glucose, mg/dL	128		93	
HbA_1c_, %	6.7	6.3	4.5	5.0
TGs, mg/dL	292	333	124	105
LDL-C, mg/dL	47.6	21.4	121.2	127.0
HDL-C, mg/dL	31	21	57	62
AST, U/L	37	52	35	36
ALT, U/L	57	78	64	58
GGT, U/L	72	224	154	163
NAFLD fibrosis score*^a^*	−1.669	−3.477	0.281	0.33
HFF, %	20	<5%	21	11
LSM, kPa	20.3 (IQR 5.3)	11.5 (IQR 16)	27.30 (IQR/M 66%)	∅
CAP, dBb/m	299 (IQR 39)	128 (IQR 10)	270 (SD 12)	∅
SSM, kPa	∅	∅	43.3 (IQR 7.0)	∅
OADs	Metformin, dapagliflozin, pioglitazone	Metformin, dapagliflozin	Metformin	Metformin
Incretin analogue	Semaglutide 1 mg/wk	Tirzepatide 15 mg/wk	Semaglutide 1 mg/wk	Tirzepatide 15 mg/wk
Insulin	Glargine 58 IU Aspart 58 IU	Glargine 20 IU Aspart ∅	∅	∅
Lipid-lowering medication	Rosuvastatin, ω-3 acid ethyl esters	Rosuvastatin, ω-3 acid ethyl esters	∅	∅

Abbreviations: ALT, alanine transaminase; AST, aspartate transaminase; BMI, body mass index; CAP, controlled attenuation parameter; GGT, γ-glutamyltransferase; HbA_1c_, glycated hemoglobin A_1c_; HDL-C, high-density lipoprotein cholesterol; HFF, hepatic fat fraction (as per latest available magnetic resonance scan); IQR, interquartile range; LDL-C, low-density lipoprotein cholesterol; LSM, liver stiffness measurement; MASLD, metabolic dysfunction–associated steatotic liver disease; NAFLD, nonalcoholic fatty liver disease; OADs, oral antidiabetic drugs; SGLT2i, sodium-glucose transporter 2 inhibitor; SSM, spleen stiffness measurement; TGs, triglycerides.

^*a*^NAFLD score correlated fibrosis severity: values less than −1.455 indicate F0-F2 (no significant fibrosis); values between −1.455 and 0.675 indicate indeterminant score (significant fibrosis cannot be ruled out); values greater than 0.675 indicate F3 to F4 (significant fibrosis) (6).

### Case 2

The individual was born to consanguineous parents and was suffering from nystagmus, visual developmental delay, and photophobia. Due to progressing visual loss, severe obesity with early childhood onset with continuing weight gain and acanthosis nigricans, he underwent genetic testing for AS, confirming the suspected diagnosis. Afterward, he entered outpatient care in our specialist pediatric clinic at age 15 years, with a BMI of 46.8 (>99.9th percentile; *Z* score: 2.97). There, he was treated with diet and physical exercise with modest success, followed by trials of liraglutide and semaglutide in off-label use without noteworthy success (lowest BMI 46.1 kg/m^2^ from a maximum of 47.8 kg/m^2^ at age 18 years to 46.3 kg/m^2^ at the time of transition to our adult clinic (see Fig. 1). He was also treated with transdermal testosterone and oral levothyroxine due to hypogonadism and hypothyroidism, respectively.

Type 2 diabetes mellitus was well controlled, with an HbA_1c_ of 4.5% on metformin 1000 mg twice a day and semaglutide 1 mg once weekly. However, he showed signs of advanced chronic liver disease in the presence of MASLD with clinical, radiological, and laboratory signs of portal hypertension as indicated by splenomegaly (15.5 cm in longitudinal diameter on a previous ultrasound), thrombocytopenia (140 G/L), and elevated von Willebrand factor antigen and activity (222% and 216%). The liver stiffness measurement (LSM) by vibration controlled transient elastography was 27.3 kPa and the continuous attenuation parameter was 270 dB/m, indicating advanced fibrosis and moderate steatosis, respectively (see Table 1). Spleen stiffness measurement (SSM) showed 43.3 kPa, suggesting the presence of portal hypertension. For further baseline characteristics, see Table 1.

## Results

Both individuals were switched from previous therapy with semaglutide 1 mg/week (see Table 1) and started on tirzepatide 2.5 mg/week subcutaneously and uptitrated as per standard titration regimen.

**Figure 2.**
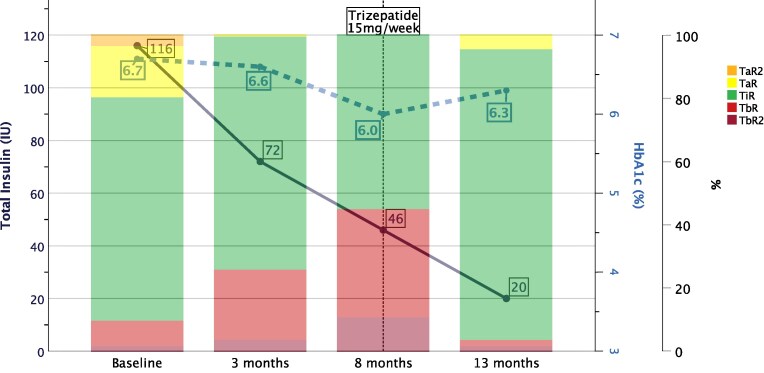
Depiction of insulin demand (IU, continuous line, scale on the left), glycated hemoglobin A_1c_ (HbA_1c_) (%, dashed line, scale on the right), and glycemic range indicator bars at baseline and after starting tirzepatide in case 1. On reaching the maintenance dose of 15 mg once weekly, a reduction of 83% (−96 IU) of total insulin requirements (ie, prandial and basal insulin) in comparison to baseline was achieved. Color-coded glycemic range indicator bars depict how much time the patient's glucose levels spend within a certain glucose range in proportions of 100% at each respective visit. Orange represents the time with glucose readings greater than 250 mg/dL (time above range 2, TaR2); yellow the time with readings between 181 and 250 mg/dL (time above range, TaR), green the time in the target range 70 to 180 mg/dL (time in range, TiR), light red the time with readings between 54 and 69 mg/dL (time below range, TbR), and dark red less than 54 mg/dL (time below range 2, TbR2). Each bar represents continuous glucose monitoring data of 14 days from a FreeStyle Libre device.

**Figure 3.**
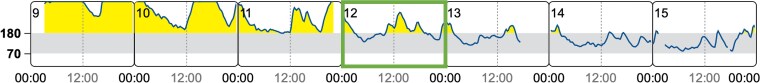
Glucose profile of case 1 over a 7-day period with readministration of tirzepatide on day 4 (marked with a box) after a period of nonavailability of the drug for approximately 6 weeks. Glucose levels were measured with a FreeStyle Libre continuous glucose monitoring system. The glucose profile rapidly normalizes after reinitiation of tirzepatide therapy on day 4 (marked with a box). Shading between 180 and 70 mg/dl indicates glucose target range. Graph based on output from LibreView (Abbott), a cloud-based glucose management system.

The first patient, aged 21 years at the time of tirzepatide initiation, was followed up until month 18 after starting tirzepatide and reached the maintenance dose of 15 mg once weekly after 6 months of standard uptitration. His weight loss was −28 kg (113.6 kg to 83.0 kg, −26.9%; resulting in a BMI change from 42.2 to 30.9 kg/m^2^) (see Fig. 1); HbA_1c_ decreased by −0.4% (6.7% to 6.3%); in addition the weight loss was accompanied by a considerable reduction in daily insulin doses from initially 116 IU/day (1 IU/kg body weight) to 20 IU/day (0.23 IU/kg body weight; 58 to 20 IU insulin glargine, −65.5%; 58 to 0 IU prandial insulin, −100%) while maintaining oral antidiabetics (metformin and dapagliflozin, with pioglitazone discontinued after ∼3 months). Hyperphagia improved according to the patient and his caregivers. He was monitored by a continuous glucose monitoring system (CGM, Abbott Libre 2), and parameters of glycemic outcomes improved continuously; however, due to the accelerated weight loss on tirzepatide, avoiding hypoglycemia was challenging, with time below range (TbR) steadily increasing despite rapid and considerable reductions in insulin doses including discontinuation of prandial insulin (Fig. 2). The coefficient of variation, indicating glycemic variability, improved steadily during the course of time with an initial value of 43.5% progressing to 29.0% at month 13. Also, hepatic steatosis, with a previous fat fraction of 20%, resolved as confirmed by magnetic resonance imaging (MRI) (see Table 1). Initially, a discrete but self-limiting increase of transaminases was observed (peak at 8 months: aspartate transaminase [AST]: 74 U/L, alanine transaminase [ALT]: 147 U/L). Fasting triglyceride (TG) levels worsened (+14.0%, 292 mg/dL to 333 mg/dL), while non–high-density lipoprotein cholesterol improved (106 mg/dL to 88 mg/dL) under stable lipid-lowering medication. Prior to the latest follow-up, tirzepatide was unavailable for 6 weeks, resulting in deterioration of blood glucose control, without weight regain. However, after reinitiating tirzepatide glucose control was reestablished after 1 day, surprisingly rapidly, as illustrated in Fig. 3. Despite mild hypoglycemia due to very rapid weight loss and improved insulin sensitivity with concomitant insulin therapy, no further adverse events occurred in this case.

The second patient, aged 20 years at the time of tirzepatide initiation, was followed-up for 9 months since the start of tirzepatide, with stable maintenance doses of 15 mg of tirzepatide reaching 4 months before the latest follow-up. He showed a weight reduction of −9.5 kg (132 kg to 122.5 kg; −7.2%; resulting in a BMI change from 46.3 kg/m^2^ at baseline to 42.9 kg/m^2^ at latest follow-up; see Fig. 1. Hyperphagia improved according both to the patient and his caregivers. MRI studies showed an improvement of the hepatic fat fraction from 21% to 11% (see Table 1). Nevertheless, MR elastography confirmed previous vibration controlled transient elastography results and showed progredient LSM consistent with advanced fibrosis. Further, MRI examinations showed progressive splenomegaly with a longitudinal spleen diameter of 17.4 cm (compared to 15.5 cm on a previous ultrasound 20 months before). HbA_1c_ changed from 4.5% at baseline to 5.0%. Lipid profiles showed mild improvements (−15.3% in TGs; 124 mg/dL to 105 mg/dL). No CGM system was used in this case since no insulin treatment was necessary. No adverse events occurred in this individual.

## Discussion

The efficacy and safety of GLP1 agonists have been investigated in a cohort study of people living with AS, showing moderate efficacy with regard to weight loss and blood glucose control compared to the general population (3). GLP1 agonists are therefore recommended to be considered for weight loss in people with AS (1). Notably, therapeutic trials with the GLP1 mono agonists liraglutide, dulaglutide, and semaglutide in our cases with AS showed only moderate efficacy with regard to weight control, which is comparable to the previously published cohort study in AS (3), with weight effects unsustained in both our cases.

However, to date, no reports regarding the use of tirzepatide in this rare and special patient population have been published. Here, we show that tirzepatide markedly improves body weight, MASLD, insulin resistance, and hyperphagia in people living with AS.

In our 2 case studies, the weight loss effects of the dual GLP1/GIP agonist tirzepatide has shown effects comparable to the previously published trials in non-AS patients, with weight reductions greater than 25% seen in 15% of participants in the SURMOUNT-2 trial, compared to −26.9% and −7.2% in our cases, after a minimum observation period of 9 months (7). The varying effect sizes between the 2 cases could be influenced by the differences in starting weight, timing of the follow-up intervals, and the total observation period.

Massive reductions in insulin doses in case 1 suggest resolution of insulin resistance, which can be more pronounced in people with AS than in controls (8). Furthermore, tirzepatide demonstrated that it effectively improved insulin resistance in patients living with type 2 diabetes (9). In case 1, insulin sensitivity rapidly improved due to very rapid weight loss and improvements in hyperphagia. This resulted in mild but recurrent hypoglycemia under insulin therapy. Therefore, considerable reductions and rapid adjustments in insulin doses should be anticipated when starting tirzepatide in individuals living with AS and diabetes mellitus on concomitant insulin therapy. In this case, close meshed control intervals during the uptitration phase of tirzepatide with biweekly to monthly visits are merited to avoid hypoglycemia. Furthermore, patients and caregivers should be instructed to be mindful of below-target fasting and postprandial glucose readings and be instructed to make adjustments to the insulin doses in close contact with the treating center. Using a CGM device with alarm function and the possibility of remote monitoring of CGM data would also help mitigate hypoglycemic events in this context.

Resolution of liver fat content was demonstrated in both individuals, comparable to the effects demonstrated in the SYNERGY-NASH trial (10). Notably, in case 1, growth hormone therapy was established at age 19 for potential benefits regarding hepatic steatosis reported in the literature (5) and continued concomitantly throughout tirzepatide therapy. This could have led to a potential overestimation of the positive effects on liver steatosis in this case (11). In the SURMOUNT-2 trial (7), fasting TGs were reduced by 27.1%, whereas no consistent reductions were seen in our 2 patients (+14.0% in case 1 patient; −15.3% in case 2 patient). However, because of the small sample size and the fact that fasting TGs are quite volatile and mostly influenced by recent dietary habits, we cannot make any definitive conclusion on the effectiveness of tirzepatide on circulating TGs in our cases. Notably, glucose was well controlled in both individuals with an HbA_1c_ below 6.5%, so it is unlikely that glucose metabolism significantly altered TGs.

Further research is needed to better understand the underlying mechanisms mediating the potentially higher efficacy of dual GLP1/GIP agonists over GLP1 mono agonists in this patient population. Tirzepatide has been demonstrated to display GIP agonism to a greater degree compared to GLP1 agonism (12); however, whether the effects in AS might be mediated by lower levels of GIP has not been investigated before, as to our knowledge, there are no reports investigating GIP and GLP1 signaling pathways in AS nor *ALMS1* mice.

However, it has been demonstrated that a loss of function of the *ALMS1* gene in adipose tissue or preadipocytes recapitulates the metabolic phenotype of a global *ALMS1* loss of function, suggesting that AS is likely caused by adipose tissue failure to expand in size and buffer a chronic positive energy balance (13-15). In contrast to GLP1 mono agonists, tirzepatide has been shown to have GIP receptor–regulated adipose tissue-specific effects, through which it improves adipose tissue function and metabolic flexibility (ie, enhanced glucose and lipid clearance in the fed state and augmented lipid release under fasting conditions) (16). Therefore, tirzepatide's adipose tissue action could lead to a potential reversal of adipose tissue failure observed in AS, perhaps explaining why tirzepatide has greater clinical efficacy in AS compared to GLP1 mono agonists. Furthermore, the more potent anorexic effects of tirzepatide compared to GLP1 mono agonists likely lead to amelioration in hyperphagia and a negative energy balance, which unloads adipose tissue, thereby improving ectopic lipid deposition and lipotoxicity.

## Conclusion

Although the small sample size, absence of a control group, and unclear definitive mechanism of action in AS are notable limitations, the presented cases underscore that tirzepatide shows great clinical effectiveness with regard to weight control, MASLD, and insulin resistance in 2 male patients with the rare monogenetic obesity syndrome AS characterized by marked hyperphagia. Further studies in larger cohorts are needed to confirm our findings. However, our data suggest that in AS when GLP1 mono agonists do not lead to sustained weight loss, a switch to the dual GIP/GLP1 agonist tirzepatide should be considered, especially when obesity-related comorbidities are present.

## Data Availability

Restrictions apply to the availability of some or all data generated or analyzed during this study to preserve patient confidentiality or because they were used under license. The corresponding author will on request detail the restrictions and any conditions under which access to some data may be provided.

## Abbreviations

ALT: alanine transaminase
AS: Alström syndrome
AST: aspartate transaminase
BMI: body mass index
CGM: continuous glucose monitoring
GIP: glucose-dependent insulinotropic peptide
GLP1: glucagon-like peptide 1
HbA_1c_: glycated hemoglobin A_1c_
LSM: liver stiffness measurement
MASLD: metabolic dysfunction–associated steatotic liver disease
MRI: magnetic resonance imaging
NAFLD: nonalcoholic fatty liver disease
OADs: oral antidiabetic drugs
SSM: spleen stiffness measurement
TbR: time below range
TGs: triglycerides
